# How to Define Spacing Among Forest Trees to Mitigate Competition: A Technical Note

**DOI:** 10.3390/biology14030296

**Published:** 2025-03-15

**Authors:** Khodabakhsh Zabihi, Vivek Vikram Singh, Aleksei Trubin, Nataliya Korolyova, Rastislav Jakuš

**Affiliations:** 1 Faculty of Forestry and Wood Sciences, Czech University of Life Sciences Prague, Kamýcká 129, Praha-Suchdol, 165 00 Prague, Czech Republic; trubin@fld.czu.cz (A.T.); jakus@fld.czu.cz (R.J.); 2 Institute of Forest Ecology, Slovak Academy of Sciences, Štúrova 2, 960 53 Zvolen, Slovakia; korolyova@fld.czu.cz

**Keywords:** Norway spruce (*Picea abies*), sap flow, tree density, ecophysiology, forest thinning, silviculture practices, tree plantation distancing

## Abstract

Establishing an optimum range of inter-species spacing that reduces competition among trees and mitigates the effects of drought is a critical yet complex challenge in forest management. Stand density plays a crucial role in forest functioning by regulating resource allocation within individual trees. Higher stand densities have been shown to reduce sap velocities, indicating intensified competition for water and other resources. However, determining the precise spacing that minimizes competition while maintaining ecosystem balance remains unclear. In this study, conducted in temperate Norway spruce forests at an altitude range of 400–500 m in the Czech Republic, we propose a novel technique to define tree spacing that reduces competitive interactions. We used xylem sap flow residuals of an ordinary least square (OLS) regression model to filter out the effects of elevation and diameter at breast height (DBH) on field-measured sap flow for 101 planted Norway spruce trees with a DBH range of 40 ± 5 cm (≈90–100 years old). The model residuals allowed us to account for the most important driver of sap flow variability: tree density and its underlying effects on individual tree traits. To minimize the confounding effects of temporal and spatial variability, we used twelve consecutive daily measurements of sap flow (6 a.m. to 6 p.m.) taken at the start of the growing season. By constructing an experimental variogram, we quantified sap flow variability as a function of tree spacing. The results showed a steady sap flow pattern at tree densities of 12, 11, and 10 trees per 314 m^2^ (equivalent to 350 ± 32 trees per hectare), corresponding to inter-tree spacing measurements of 5.12 m, 5.34 m, and 5.60 m, respectively. These findings suggest that when the *N* number of trees (median) per unit area (*A*) is in equilibrium with resource availability, increasing or decreasing the *n* number of trees may not significantly change competition levels (*A; f(A) = N ± n*). The size or deviation of *n* depends on the area to define the minimum and maximum thresholds or tolerance capacity for the number of trees allowed to be in the area. This technique—using a variogram of sap flow residuals to determine tree spacing—can be periodically applied, such as every 10–15 years, and adapted for different elevation gradients (e.g., within 100 m intervals). It offers a practical tool for forest managers and policymakers, guiding thinning and planting strategies to enhance forest resilience in the face of water-stress conditions.

## Simple Summary

Defining the optimal spacing between tree species to reduce competition and thus mitigate drought effects is essential but highly complex. The equilibrium in terms of the number of trees, where competition for shared resources like water, nutrients, and light is minimized, depends on various environmental and tree-related factors. This study, conducted in the temperate Norway spruce forests of the Czech Republic at an altitude of 400–500 m, introduces a new method for determining ideal tree spacing to reduce competition. Here, we filtered out the effects of elevation and diameter at breast height on field-measured xylem sap flow for 101 Norway spruce trees, aiming to account for the most important driver of sap flow variability: tree density and its underlying effects on individual tree traits. We found that when the *N* number of trees (median) per unit area has the least competition pressures, adding or removing the *n* number of trees may not significantly change the situation, thus affecting competition among trees. The tolerance capacity (*n*) is a function of area (A) that determines the permissible deviation from N. Our technique offers a new tool for forest managers and policymakers, helping them make informed decisions about thinning and plantation strategies based on elevation gradients.

## 1. Introduction

Norway spruce (*Picea abies* [L.] Karst.) is one of the most important conifer tree species of the Northern Hemisphere, dating back to the early Holocene around 9500 years ago [1,2]. Adapted to cool, moist climates, its range spans from central and eastern Norway through Fennoscandia, the Baltic states, Belarus, and Russia, and extends into Central and Southeastern Europe. It has a long history of plantations to provide socio-economic and ecological services. Considered a large coniferous species, it typically undergoes a lifespan of approximately 200–300 years, attaining a height of 50–60 m and a trunk diameter of up to 150 cm as it matures [3,4,5,6].

Competition among trees is a fundamental ecological process influencing growth, survival, and resource allocation. In dense forest stands, competition for water, nutrients, and light can lead to increased stress, particularly in drought-prone regions. The shallow root system of Norway spruce makes it vulnerable to wind disturbances and summer droughts, particularly when grown in a dense stand [2,4,6,7,8]. Therefore, mature spruce trees require more space to access soil moisture during the dry seasons to mitigate the competition pressures against neighboring trees in using shared resources. In a diverse forest ecosystem, competition among trees is a function of species, size, and distance among neighboring trees [9]. An initial wide spacing and thinning have been recommended for Norway spruce stands to produce a merchantable wood volume on a shorter temporal scale [10,11]. The wider initial spacing became a trend in silviculture practices that improved individual tree growth and lowered establishment costs, aimed at decreasing the frequency of thinning [12]. However, extensive space among trees may cause more exposure of interspace non-tree gaps to solar radiation, which increases evapotranspiration during the dry seasons [8,13,14,15]. Although wider plantation spacing is reported to enhance diameter growth and increase survival rates, it may also lead to greater stem taper, which can reduce the merchantable volume of individual trees [16]. Thus, defining a range of spacing/distance among trees that could mitigate the effects of competition is still a persisting challenge. For example, can a particular technique be periodically employed to define optimum spacing among trees while a tree ages within a stand, which necessitates an increase in spacing?

Xylem sap flow can indicate tree water uptake and transpiration functions and thus assess forest health and responses against water-stress stimuli [8,15,17,18]. Although several studies have contributed to understanding influential factors on sap flow variabilities, information about the characterizing spacing among trees based on sap flow measures has yet to be investigated. The influential environmental factors on sap flow were found to be soil water potential [8,19,20,21,22], elevation [15], solar radiation and vapor pressure deficit [8,19,20,22], photosynthetically active radiation (PAR) [22,23], air temperature [22], slope [24,25], and aspect and soil texture [26].

Some of the tree-related factors that were influential on sap flow were angles of tree deviations from the axis, stem moisture, the internal temperature of trunks, and the normalized difference vegetation index (NDVI) [27]. The NDVI is highly related to leaf biomass [15]. Sap flow was highly correlated with tree density [8,15,23,26], a reverse estimate of tree spacing. Thus, sap flow can be used to assess competition among trees for using shared resources, such as soil moisture contents, if all influential factors on competition, except tree density and density-driven factors, are either kept on hold or have equal impacts on trees within a stand. For example, variations in sap flow and the growth of spruces were explained by a competition index (CI), a function of distance, tree diameter, and the azimuthal direction of neighboring trees from a focal tree [28].

We aimed to (1) find the number of spruce trees per given area that could mitigate the competition pressures; (2) compare our findings of characterized spacing (or, conversely, density) among trees to National Forest Inventory (NFI) data documented in other countries in Europe; and (3) provide a technique to define a required range of spacing among trees that may better inform future silviculture practices and possible tree plantation strategies.

## 2. Materials and Methods

### 2.1. Study Area and Field Measurements

**Figure 1:**
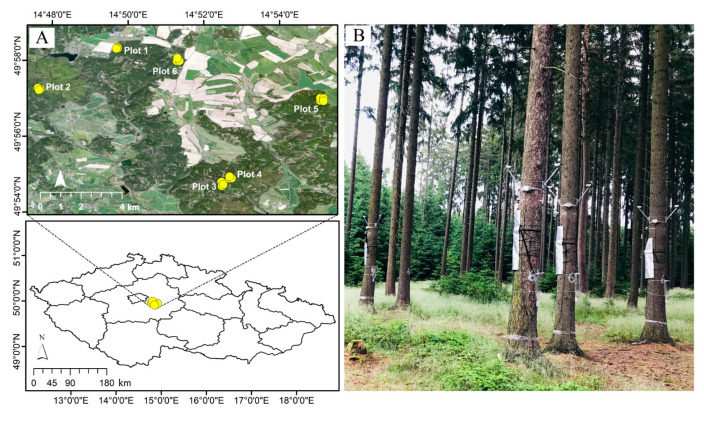
(**A**) Study area with 101 Norway spruce tree positions (yellow circles) within 6 plots (24 subplots: 1A–D, 2A–D, 3A–D, 4A–D, 5A–D, and 6A–D) at the university forest enterprise in Kostelec nad Černými Lesy, Czech Republic. (**B**) A picture of the experimental setup from a subplot with infrared temperature and sap flow sensors installed at the upper and lower parts of trunks, respectively. The figure is adapted from Zabihi et al. (2023) [15].

The study focused on six established plots within a Norway spruce-dominated plantation forest in Central Europe (a university forest enterprise in Kostelec nad Černými Lesy, Czech Republic; Figure 1). This study is a continuation of our previously published work [15] using datasets collected from the same plots in the same study area.

The vegetation in the study area is predominantly Norway spruce, with smaller proportions of pine and beech. The Czech Republic lies in the temperate climate zone. The climate in the study area is relatively drier and warmer in summer, with a growing season ranging from 150 to 160 days and a mean annual temperature and precipitation of 7–7.5 °C and 600 mm, respectively [29,30]. The terrain is relatively flat, with the slope ranging from 0.8° to 6° and elevation ranging from 407 to 492 m. Each plot consisted of four subplots, i.e., A, B, C, and D, with a stand of 8–10 Norway spruce trees having an average DBH of 40 cm (SD = 6 cm; ≈ 90–100 years old) (Supplementary Material). Plots 3 and 4 were established within proximity at 430 m, whereas distances among other plots (1, 2, 5, and 6) and from those two plots (nos. 3 and 4) were relatively far, ranging from 2 to 9 km (Figure 1). Subplots within plots were spaced at a 40 to 250 m distance range. The prevailing mineral bedrock across the study area was granodiorite covered with luvisol [31]. The xylem sap flow was measured by EMS 81 sensors (EMS Brno, Brno, Czech Republic), based on the trunk heat balance method (THB) [8,32], installed at a height of 2 m in the year 2018. The longitude (X-coordinate), latitude (Y-coordinate), and elevation (m) of each tree position were also recorded by a Global Positioning System (GPS). The GPS-related errors in tree positions were later fixed with the ArcGIS collector app (ESRI Inc., Redlands, CA, USA) installed on a smartphone, with high spatial resolution (20 cm) Unmanned Aerial Vehicle (UAV) images that were underlying the tree positions of each plot. We used an average sum of 12 h (6 a.m.–6 p.m.) sap flow measurements for twelve consecutive days, 15–26 April 2019, for 101 trees (daily sum on average). The daily air temperature (6 a.m.–6 p.m.) was 16 °C on average (SD = 4 °C) for the twelve days. The soil water potential (*Ψw*) was measured at the topsoil at a 10 cm depth (Teros 21, Meter group, München, Germany) using five sensors in each subplot as a single measurement. We calculated tree density within the 314 m^2^ area surrounding every tree using a 10 m radius buffer size from a focal tree position to account for the potential influence of neighboring trees on the focal tree transpiration.

The trees were counted manually in the ArcGIS Desktop (ver. 10.8.1) (ESRI Inc., Redlands, CA, USA, [33]) using very high spatial resolution (20 cm) UAV images that were underlying the tree positions of each plot. The sap flow measurements for a single day on 19 April 2019 and the rest of the measurements, such as tree density, DBH, and elevation, were also used for 37 out of these 101 trees in our previous work [15]. However, our previous study was only aimed at understanding the main drivers of sap flow variability.

### 2.2. Estimated Spacing Among Trees Using Tree Density Within the Buffer

We assumed trees were distributed in equally spaced grid patterns to calculate distance/spacing among trees within a defined 10 m buffer (Table 1). We did not measure distances among trees within every buffer to make an average distance, as averages were found to be very sensitive to extreme values or outliers. In addition, the crowns of some neighboring trees were intercepted, and thus, measuring distances between them was error-prone.

We took the squared root of the area of defined buffers (A = 314 m^2^) divided by the number of trees within the buffers (tree density) to calculate the distance among Norway spruce trees as follows:

(1) $$D=\sqrt{\frac{314}{n}}$$

where D stands for equal distances among rows and columns of an *n* number of trees if trees are assumed to be spatially distributed in regular grid patterns within a 10 m radius buffer.

### 2.3. Controlling the Effects of Nuisance Variables on Sap Flow Before Developing the Models

As both individual tree traits and several environmental variables may affect sap flow, we approached it differently to control the effects of these factors while only assessing the effects of tree density and its underlying effects (individual tree traits) on sap flow. For example, using sap flow measures at the beginning of the growing season (May–October) allowed us to assess sap flow variations among trees in average weather conditions where trees were not water-stressed [15]. A shorter temporal measure of sap flow for 12 consecutive days within the extent of our study, considered as a regional scale (Figure 1), also allowed us to minimize the effects of the temporal and spatial variability of climate variables, e.g., temperature and precipitation, on sap flow measures [15]. The area’s flat terrain allowed us to avoid the effects of slope and aspect on sap flow. The influence of soil water potential on sap flow was found to be negligible as its variability was limited across subplots [15]. Also, none of the stems deviated from the axes. Trees were found to be almost even-aged as the variability of DBH was limited (*µ* ± *σ* = 40 ± 6 cm; ≈90–100 years old). However, we included DBH among other variables (elevation and tree density) in the proposed regression models to consider the effects of DBH on sap flow variability (Table 2).

### 2.4. Regression Diagnostics

All steps of regression diagnostics and developing models were performed according to Zabihi et al. [15] using the GeoDa open-source software (ver. 1.18.0) [34]. We initially ran Pearson’s correlation test among all variables in R (ver. 4.1.2; R Core Team, Vienna, Austria, 2021; Table 3) to avoid including highly correlated explanatory variables (r ≥ 0.7) in the proposed models.

We developed a spatial weight matrix employing the first-order Queen Contiguity weight algorithm (GeoDa) [34] on a vector shape file of 101 tree positions [33]. The developed spatial weight matrix was used to calculate Moran’s *I* to examine the spatial dependency (autocorrelation) of ordinary least square regression (OLS) model residuals. If OLS model residuals were spatially autocorrelated (significant *p*-value for Moran’s *I*), spatial regression (lag or error) models were used to take the spatial autocorrelation into account. The spatial weight matrix was additionally used to assess the spatial autocorrelation in sap flow measures as well as in model covariates, tree density, elevation, and DBH. The Akaike Information Criterion (AIC) [35] was used to evaluate the performance of the models. If the AIC difference (∆AIC) between spatial lag and error models was less than or equal to two (∆AIC ≤ 2), those models were considered equally performing models. The Jarque–Bera and Breusch–Pagan tests were used to assess the normality and heteroskedasticity of the errors, respectively.

### 2.5. Filtering out the Effects of Elevation and DBH on Sap Flow

As our samples were collected within a 100 m elevation range with variability in DBH, although limited, the effects of these influential factors on sap flow (Table 2) needed to be removed before assessing the tree density effects on sap flow. We took advantage of the residuals of a regression model to filter out the effects of elevation and DBH while capturing the effects of tree density and possibly other density-driven factors, e.g., individual tree traits, on sap flow residuals. We modeled sap flow as a function of elevation and DBH to remove the effects of tree density and the underlying density-driven factors in the model residuals, considering them as unmeasured variables.

### 2.6. Experimental Variogram

A variogram is used to represent the variability among the target value of sampled points as a function of distance. We plotted the variance of sap flow residuals, at which elevation and DBH effects on sap flow were removed, as a function of distance among trees, calculated from the tree density within the buffers (Table 1).

Our sample locations were at different plots (spatial locations) with diverse tree densities within the buffers from plot to plot. For example, plot 1 had a tree density of 10–14, while plot 2 had a tree density of 8, 9, and 11–13 (Supplementary Material). Although we plotted the sap flow variability against distances, our approach was different from the commonly used variogram, measuring the variance of a target value by increasing distance from a focal point. We proposed a situation to understand how the equilibrium state of using shared resources among trees, such as water, changes if an augmented number of trees is randomly located within an area. In other words, the fewer the fluctuations in sap flow variability, by increasing the number of trees within 314 m^2^, the more stable the situation, defining the equilibrium state of using shared resources.

## 3. Results

### 3.1. Proposed Regression Models

None of the assumptions for model residuals, including normal distribution, heteroskedasticity, and multicollinearity, were violated in any of the proposed models (Table 4).

In model no. 2, residuals were not spatially autocorrelated (Moran’s I *p*-value = 0.079; Table 3) for the OLS model. Thus, the OLS model was used to remove the elevation and DBH effects from the field-measured sap flow. The assumption of spatial dependency of model residuals was violated in models no. 1 and 3 (full model), and therefore, spatial regression models were used to take the spatial autocorrelation into account (Table 3). We used the residuals of model no. 2 for the next steps of the analyses, while models no. 1 and 3 were only developed for understanding the influential drivers of sap flow variability.

### 3.2. Spatial Autocorrelations in Sap Flow Drivers, Sap Flow, and Sap Flow Residuals

**Figure 2:**
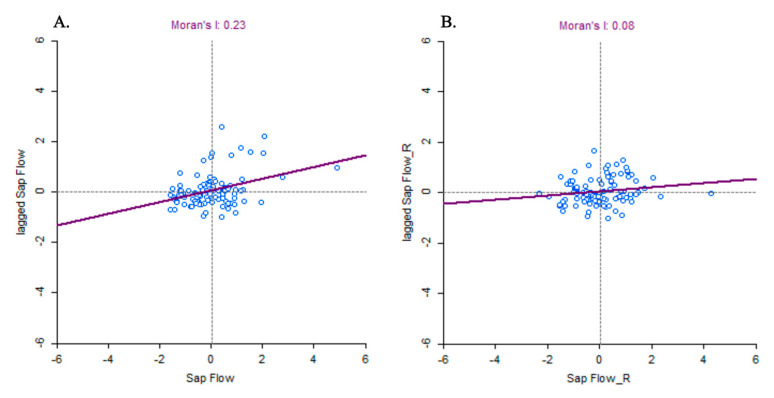
Moran scatter plot: (**A**) standardized field-measured sap flow on the X-axis used to compute its spatially lagged counterparts on the Y-axis; (**B**) standardized sap flow residuals of model no. 2 (X-axis) with their spatially lagged counterparts on the Y-axis. The slopes of the solid red lines correspond to Moran’s *I*, with associated values of 0.23 and 0.084 for field-measured sap flow and sap flow residuals of model no. 2, respectively.

Sap flow, tree density, and elevation were spatially autocorrelated (Moran’s *I* = 0.23, 0.28, and 0.84, respectively; pseudo-*p*-value with 999 permutations = 0.001; Figure 2A for sap flow), whereas DBH was not (Moran’s *I* = 0.056; pseudo-*p*-value with 999 permutations = 0.142). After running model no. 2 to remove elevation and DBH effects from field-measured sap flow, the sap flow residuals became spatially independent (Moran’s *I* = 0.08, pseudo-*p*-value with 999 permutations = 0.084; Figure 2B). The observed patterns indicate that the spatial autocorrelation effects on sap flow were removed from the model residuals by elevation effects in model no. 2, as elevation was strongly spatially autocorrelated.

### 3.3. Experimental Variogram

**Figure 3:**
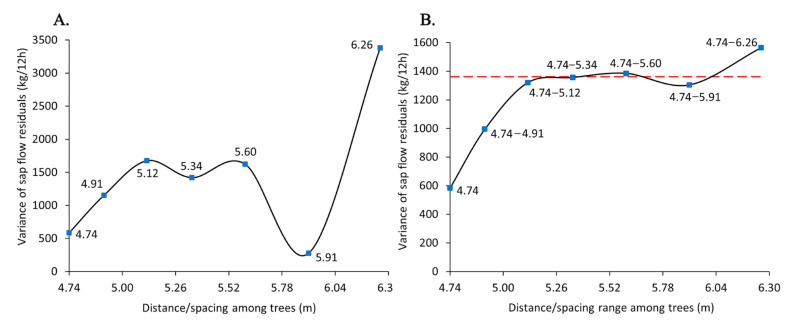
Experimental variograms: (**A**) variance of sap flow residuals (kg/12h) as a function of distance/spacing (m) among Norway spruce trees; (**B**) variance of sap flow residuals as a function of distance/spacing range among Norway spruce trees. The variance levels off at a 5.12 m distance, followed by 5.34 m, 5.60 m, and 5.91 m distances (red dash line; sill). The x-axis is the square root of the area of defined 10 m buffers (A = 314 m^2^) divided by the number of trees within the buffers (tree density). The y-axis is the variance of the effects of unmeasured variables of tree density and the underlying density-driven factors on sap flow (variance of residuals of model no. 2) after removing the effects of DBH and elevation on sap flow.

Two types of experimental variograms were displayed: a point-to-point function of the variance of sap flow residuals at single spacing (Figure 3A) and a point-to-point function of the variance of sap flow residuals at augmented ranges of spacing, e.g., 4.74, 4.74–4.91 m, 4.74–5.12 m, 4.74–5.34 m, 4.74–5.60 m, 4.74–5.91 m, and 4.74–6.26 m (Figure 3B).

At three consecutive distances (5.12 m, 5.34 m, and 5.60 m), the variance of sap flow residuals followed the steadiest patterns with the least fluctuations compared to other distances. The variance followed an abrupt change at 5.91 m with a declining pattern at 5.60–5.91 and an increasing trend at 5.91–6.26 m. Trees in different groups of density (or, conversely spacing) were from different spatial locations as their associated plot- and subplot-designated names varied.

## 4. Discussion

### 4.1. Spacing Range to Mitigate Competition Pressure

Sap flow variability followed a steady pattern starting from 5.12 m to 5.34 m and eventually to 5.60 m spacing among trees, measured at different spatial locations within plots and subplots (Figure 1 and Figure 3). These spacings correspond to 12, 11, and 10 trees per 314 m^2^, respectively. The observed patterns suggest that the equilibrium state of an *N* number of trees per unit area (*A*), with minimum restrictions from neighboring trees for accessing shared resources, may remain unaltered even with an increase or decrease in an *n* number of trees (*N ± n*) depending on the area (*f(A)*), as follows:

*A*; *f*(*A*) = *N* ± *n*(*A*) (2)

where *n* defines the maximum and minimum threshold number of trees that can be tolerated by the *N* (median) number of trees per area (*A*) without having significant negative impacts on the competition among trees in equilibrium. The tolerance capacity (*n*(A)) is a function of area *A* that defines the allowable deviation from N, as follows:

(3) $$n(A)=\alpha \cdot A^{\beta }$$

where α is a proportionality constant, representing how resource availability or competition scales with area, and β is a scaling exponent that determines the relationship between *A* and the deviation *n*. For example, β = 0.5 might suggest that tolerance grows with the square root of the area, whereas Β = 1 implies a linear relationship. Thus, *f*(*A*) defines the range of acceptable tree density, where

$$f\min(A)=N-\alpha {\cdot A}^{\beta }$$

$$f\max(A)=N+\alpha \cdot A^{\beta }$$

**Figure 4:**
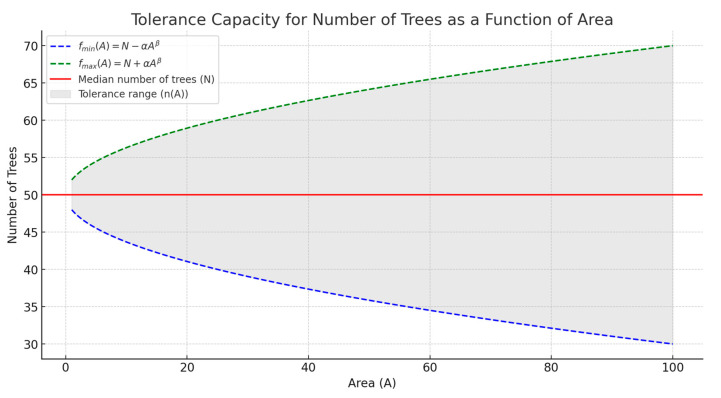
Tolerance capacity for the number of trees as a function of area. Tree density thresholds expand or contract with increasing unit area (A). The red line represents the median number of trees, e.g., 50. The blue and green dashed lines indicate the minimum (*f*_min_(A)) and maximum (*f*_max_(A)) thresholds for the number of trees as the area (A) increases. The shaded region shows the acceptable tolerance range for the number of trees, which expands as the area grows.

α and β can be adjusted based on ecological parameters, such as resource-sharing dynamics or competition intensity. This formula (*f(A) = N ±* $\alpha \cdot A^{\beta }$) can model how tree density thresholds expand or contract with increasing unit area (Figure 4).

Sap flow at below 5.12 m and above 5.60 m spacing among trees experienced more unsteady patterns with high sensitivity to the size of spacing. The high tree density and, thus, the elevated competition pressure at below 5.12 m spacing among trees may cause the sap flow variability to change with different sizes of spacing: 5.12 m vs. 4.91 m vs. 4.47 m. On the other hand, a wide space above 5.60 m might create large interspace gaps in the stand canopy, allowing a higher load of incoming solar radiation and, consequently, increased evaporative demands. The high evaporative demand may increase the water requirements by trees within a stand, thus causing high variability in sap flow.

The 10–12 trees per 314 m^2^ (5.12–5.60 m spacing) correspond to 318 to 382 Norway spruce trees, with a DBH of 40 cm on average, within a hectare with a median number of trees at 350 (*N ± n;* 350 ± 32 trees per hectare). Our finding of the spacing range falls within the average range of a crown diameter of about 5–6 m for coniferous species in general [36,37]. However, Krajicek et al. [38] modeled the crown diameter of open-grown Norway spruce trees, without having any competition effects, as a function of DBH and found that trees like our sampled trees with a DBH of 40 cm can reach 7.6 m in crown diameter. Thus, the average crown diameter of about 5–6 m may be used as a rule of thumb to define a minimum spacing range required to plant a spruce tree to minimize the overhanging and shading effects from branches of neighboring trees on each other in later ages. For example, Mäkinen and Hein [12] found that wide-range spacing among young Norway spruce with a stand density of 350 per hectare significantly improved the branch diameter, exceeding the round wood class B requirements in European standards, compared to tree densities of 700 and 1600 per hectare. Similarly, a study by Katrevičs et al. [39] on 50-year-old planted Norway spruce revealed that trees with low density, at 5 × 5 m^2^ spacing, resulted in a profound increase in tree diameter and height compared to higher density plantations at 1 × 3 m^2^ spacing.

### 4.2. Comparison to National Forest Inventory (NFI) Data on Tree Density

Our sampled tree density range in equilibrium (350 ± 32 trees per hectare) was found to be close to low-density forests in other regions of Europe, provided as National Forest Inventory (NFI) data. For example, 360–1280 trees per hectare are recorded in Latvia (NIF data) [39], and 231.95 (SD = 13.45) live trees per hectare are recorded in southern Switzerland (NFI4) [40]. We did not assess the susceptibility of different stand densities to biotic and abiotic stressors, e.g., pest infestations, rotting roots, and windstorms, which are currently a main concern in worldwide forests. However, several previous studies have found that lower tree density, even during the plantations and young ages, can increase tree resilience against these stressors due to greater radial increments, influencing tree resistance, and decreased intraspecific competition among neighbor trees [39,41,42,43,44,45].

### 4.3. The Introduced Technique

We introduced a novel approach of using the variogram of sap flow residuals, with existing effects of tree density, after removing the effects of other influential factors on sap flow variability as well as controlling the effects of other nuisance environmental variables. The unmeasured variables in our models, which may remain in model residuals, could be factors related to tree traits such as crown length and radius [15]; however, these factors are highly driven by tree density. For example, Thrope et al. [9] found tree traits, such as crown length and radius, as a function of neighboring tree density and height. The low stand density was also found to be an influential factor in increasing branch diameter [12], DBH, and tree height [39]. However, we removed DBH effects from the field-measured sap flow to consider similar characteristics for individual tree traits, as DBH correlates with individual tree traits such as tree height and crown size.

### 4.4. Future Silviculture Practices

Our sampled Norway spruce trees were at 407 to 492 m (~100 m) elevation ranges, which include low-altitude temperate Norway spruce forests, and we controlled the highly influential effects of elevation on sap flow. As the tree density in forests varies based on the elevation range [15,40] (Table 3) and development stages [40], our range of spacing may vary by elevation gradients, in addition to the climate variations in Norway spruce forests. As the crown diameter of trees varies by age, which is related to DBH, the minimum spacing among trees may need to be defined every 10–15 years for every 100 m elevation range. Thus, our findings could be used as a minimum required spacing range for spruce forests in the temperate zone at the 400–500 elevation range with a DBH of 40 m on average to mitigate the competition effects from neighboring trees. However, Gleason et al. [46] recommended maintaining forest stands at a lower density to improve forest resilience against droughts in all climate conditions, as competition consistently impacts tree growth response to drought. In a recent study, Bernal et al. [47] suggested thinning to promote or maintain tree growth and vigor, even during severe drought conditions, to ameliorate tree susceptibility to bark beetle-associated mortality causes.

## 5. Conclusions 

Our findings indicate that competition among Norway spruce trees is minimized at a spacing of 5.12–5.60 m, corresponding to 350 ± 32 trees per hectare. The introduced technique—using sap flow residuals through variogram analysis— provides a spatial tool for defining optimal tree spacing across elevation gradients. This method allows forest managers to move beyond traditional thinning schedules and instead implement a dynamic, data-driven approach tailored to site-specific conditions. However, the spacing for plantations may vary based on different purposes, such as timber stocking, windbreaks, wildlife, and community or recreational values. We used different approaches to control the effects of most nuisance variables on sap flow, while for some of the variables, the spatial variability was limited due to our regional-scale study. Therefore, if there is any significant variability in climate factors, such as temperature and precipitation, and site characteristics, e.g., soil texture and water availability, slope, and aspects within a 100 m elevation range, their effects on sap flow need to be considered, for example, by sampling sap flow and its driving factors at different spatial locations to take the existing spatial variability into account. As most environmental variables are spatially autocorrelated, their variability is usually limited within the extent of small regions. As trees grow, their access to shared resources is gradually minimized. Thus, a strategy of planting trees with a minimum spacing range around the average crown diameter may be initiated while repeated further thinning can be used every 10–15 years in mature stands using our introduced approach. This study not only refines our understanding of tree competition but also sets the stage for adaptive silvicultural strategies that align with the realities of spatial and temporal variabilities in the environment, including climate. By periodically recalibrating tree spacing based on real-time physiological indicators, forests can be managed with greater precision, promoting resilience against drought stress and maximizing growth efficiency.

## Figures and Tables

**Table 1:** The number of observed trees within 10 m radius buffers (tree density) used to calculate distance among trees assuming equal spacing in trees in rows and columns, and the number of observed buffers with a particular tree density.

No. of Trees Within a 10 m Radius Buffer (Tree Density)	Distance Among Trees Assuming Equal-Spaced Trees (m)	No. of Observed Buffers with a Particular Tree Density
14	4.74	9
13	4.91	13
12	5.12	24
11	5.34	25
10	5.60	13
9	5.91	7
8	6.26	10

**Table 2:** List of explanatory/independent variables (covariates) and response/dependent variables of proposed regression models.

Dates, Time	Dependent/Response Variable	Independent/Explanatory Variables (Covariates)
		Elevation (m)
15–26 April 2019, 6 a.m.–6 p.m.	Sap flow (sum on average; kg/12 h)	Tree density
		DBH (cm)

**Table 3:** Correlation matrix between response (sap flow sum on average for 12 consecutive days, 15–26 April 2019) and explanatory variables. Correlation coefficient (*r*) and determination (*r*^2^ in parentheses) and the significance of the correlation coefficient or *p*-value are provided for a one-by-one variable.

Variable	Elevation	Sap Flow	DBH	Tree Density
Elevation	1			
Sap flow	0.49 (0.24), <0.01	1		
DBH	0.31 (0.1), <0.01	0.47 (0.22), <0.01	1	
Tree density	−0.50 (0.25), <0.01	−0.43 (0.18), <0.01	−0.21 (0.04), 0.03	1

**Table 4:** Proposed regression models with associated AIC to assess model performance. The model no. 2 was used to remove the effects of elevation and DBH from the field-measured sap flow.

Proposed Model	Model no.	OLS Adjusted R^2^	OLS AIC	OLS Moran’s *I p*-Value	Spatial Lag R^2^	Spatial Lag AIC	Spatial Error R^2^	Spatial Error AIC
TreeDensity	1	0.18	1055.70	0.046	0.22	1053.37	0.22	1053.26
Elevation + DBH	2	0.33	1034.43	0.079	*-*	*-*	*-*	*-*
TreeDensity + Elevation + DBH	3 (Full Model)	0.37	1030.83	0.046	0.40	1032.19	0.41	1028.51

## Data Availability

The datasets generated during and/or analyzed during the current study are available from the corresponding author upon reasonable request.

## Supplementary Materials

The following supporting information can be downloaded at: https://www.mdpi.com/article/10.3390/biology14030296/s1, Dataset of tree attributes and Analysis.
